# Molecular Sensomics Combined with Random Forest Model Can Reveal the Evolution of Flavor Type of Baijiu Based on Differential Markers

**DOI:** 10.3390/foods13193034

**Published:** 2024-09-24

**Authors:** He Huang, Yiyuan Chen, Yaxin Hou, Jiaxin Hong, Hao Chen, Dongrui Zhao, Jihong Wu, Jinchen Li, Jinyuan Sun, Xiaotao Sun, Mingquan Huang, Baoguo Sun

**Affiliations:** 1 Key Laboratory of Brewing Molecular Engineering of China Light Industry, Beijing Technology and Business University, Beijing 100048, China; huanghe3938@163.com (H.H.); chenyiyuan_1112@163.com (Y.C.); houyaxinjy@163.com (Y.H.); m15205199608@163.com (J.H.); chen2000202208@163.com (H.C.); wujihong12@126.com (J.W.); lijinchen@btbu.edu.cn (J.L.); sunjinyuan@btbu.edu.cn (J.S.); sxt_btbu66@163.com (X.S.); hmqsir@163.com (M.H.); sunbg@btbu.edu.cn (B.S.); 2 China Food Flavor and Nutrition Health Innovation Center, Beijing Technology and Business University, Beijing 100048, China; 3 Department of Nutrition and Health, China Agriculture University, Beijing 100048, China; 4 School of Light Industry Science and Technology, Beijing Technology and Business University, Beijing 100048, China; 5 Laboratory of Food Quality and Safety, Beijing Technology and Business University, Beijing 100048, China; 6 College of Chemistry and Materials Engineering, Beijing Technology and Business University, Beijing 100048, China

**Keywords:** Baijiu, flavor type, molecular sensomics, differential marker, correlation analysis, random forest

## Abstract

Baijiu is popular with a long history and balanced flavor. Flavor type is the most widely used classification mode for Baijiu. However, the evolutionary relationships of Baijiu flavor types and the differential markers between flavor types are still unclear, significantly impacting the development of the Baijiu industry. In this study, a total of 319 trace components were identified using gas chromatography–olfactometry–mass spectrometry and gas chromatography–mass spectrometry. Among them, 91 trace components with high odor active values or taste active values were recognized as flavor components. Then random forests were conducted to screen differential markers between the derived and basic flavor types, while a principal component analysis assessed their effectiveness in distinguishing the flavor types of Baijiu. Finally, 19 differential markers (including 3-methylbutyric acid, pentanoic acid, 2-butanol, 2,3-butanediol, ethyl pro-panoate, isobutyl acetate, ethyl butanoate, ethyl hexanoate, ethyl heptanoate, ethyl lactate, ethyl 2-hydroxy butanoate, isopentyl hexanoate, ethyl nonanoate, isopropyl myristate, ethyl tetradecanoate, ethyl benzoate, 2,4-di-t-butylphenol, 2-methylbutanal and 3-octanone) were screened and proven to effectively reveal the evolution of Baijiu flavor types; these were further verified as key differential markers using addition tests and a correlation analysis.

## 1. Introduction

Baijiu is the most popular alcoholic beverage in China and has been historically and culturally relevant for more than 2000 years [1]. It has penetrated almost every aspect of social life and has become a medium to enable human beings to express their feelings and communicate with each other. According to industry statistics from the China Liquor Industry Association, in 2023, the annual output of Baijiu was 6.29 billion liters and fell by 5.1% year over year. However, the annual profit was 326.15 billion USD, accompanied by a growth of 7.5%. In general, the production capacity gradually decreased but the annual profit notably increased, which indicates that the output of mid-tier and high-end Baijiu increased. In the future, the Baijiu industry will move towards high-quality product development.

Known for its complex brewing techniques and unique flavor, Baijiu is collectively known as one of the six world-renowned distilled liquors, along with whiskey, brandy, vodka, gin and rum [1]. Baijiu is mainly composed of water and ethanol with trace components comprising about 2%. Differently from other distilled liquors, Baijiu is fermented with single sorghum or mixed grains (sorghum, glutinous rice, corn, etc.) and co-fermented with multiple microorganisms (*Bacillus*, *Microbacterium*, *Pseudomonas*, *Corynebacterium*, etc.) [2], which provide a rich material basis for the generation of trace components in Baijiu. According to the statistics of our team, it was reported that 3443 kinds of trace components have been detected in Baijiu, including 792 esters, 419 alcohols, 293 acids, 37 lactones, 384 aldehydes and ketones, 65 acetals, 217 sulfur components, 304 nitrogen components, 127 furans, 92 pyrazines, 74 heterocycles, 241 aromatics (containing benzene), 179 hydrocarbons, 136 terpenes and 83 others. These numerous and rich kinds of trace components affect the formation of various flavor characteristics of Baijiu. It would be unscientific to use a unified standard to evaluate all types of Baijiu, which is a factor affecting the standardization of the Baijiu industry. Thus, the concept of flavor type has been proposed.

The concept of flavor type was formed in 1952, at the First China Famous Baijiu Appraisal Meeting. Judges ranked Baijiu according to their own preferences and standards in the local region. This event led to the birth of the revolutionary technical categories of Baijiu: Maotai and Fenjiu pilots. It was found that, although these Baijiu varieties were famous and were of high quality, their sensory and trace components were extremely different. Therefore, the question of whether Baijiu should be classified first by flavor type and then graded was raised. The earliest definite decision on Baijiu flavor type was from the Third China Famous Baijiu Appraisal Meeting in 1979, when the concept of ‘Xiangxing (i.e., flavor type)’ was put forward. At that time, there were only four basic flavor types: qingxiangxing Baijiu, nongxiangxing Baijiu, jiangxiangxing Baijiu and mixiangxing Baijiu. Due to the unclear classification standards for the flavor types of Baijiu, many famous Baijiu products were not selected. After more than 30 years of development, integration and consolidation, eight flavor types, including chixiangxing Baijiu, texiangxing Baijiu, fuyuxiangxing Baijiu, dongxiangxing Baijiu, zhimaxiangxing Baijiu, laobaiganxiangxing Baijiu, fengxiangxing Baijiu and jianxiangxing Baijiu, have been gradually derived, forming the 12 flavor type systems of Baijiu [3].

In the early stage of development, a few representative enterprises solidified the production technologies of Baijiu, and then their production technologies were promoted in other regions. During this process, many enterprises optimized and improved traditional production technologies by adapting them to the local culture, ecological environment and eating habits. Of note, the production of Baijiu is extremely affected by water, raw materials, the ecological environment, etc. Even if the same production technology is applied to different regions, the flavor of the Baijiu produced will be quite different. Hence, the correlation of and difference between the flavors of different types of Baijiu have received more and more attention. Currently, traditional sensory evaluations of Baijiu flavor mostly rely on manual work; that is, human senses (i.e., smell, taste) are used to comprehensively evaluate the appearance, aroma, taste and typicality of Baijiu, a process which is full of subjectivity. Although the terms related to the classification of Baijiu and the evaluation of Baijiu are standardized and described in the current national standards, they are still vague and general. The material basis responsible for the difference between Baijiu flavor types is still unclear. Therefore, we could help clarify the relationship between Baijiu flavor types by revealing the differential markers of different flavor types at the molecular level and analyzing the corresponding relationship between the differential markers and sensory quality.

According to research, the flavor quality of Baijiu mainly depends on the distribution characteristic of trace components. Thus, current research has mainly focused on the mining and evaluation of trace components and flavor components in Baijiu by the application of multiple pretreatment techniques combined with detection techniques. Of note, the vacuum-assisted sorbent extraction (VASE) method is simple to operate and largely automated; in addition, it has a large adsorption phase volume, a high extraction efficiency and a high degree of sensitivity. It is suitable for identifying and analyzing trace components in complex liquid matrices (as shown in Figure S1) [4]. However, there are few reports on its application for the analysis of trace components in Baijiu. Furthermore, the dataset of trace components in Baijiu is quite large, but not all trace components directly contribute to the sensory attributions of Baijiu. Therefore, it is necessary to use machine learning and molecular sensomics to reduce the dimension of the dataset and further screen and verify the key flavor components in Baijiu. Researchers [5] used the random forest model, a partial least squares discriminant analysis and a Spearman correlation analysis to screen the different components in Baijiu of different ages from 98 kinds of volatile compounds. The results showed that ethyl oleate was found to be the best single age marker. In previous studies [6], random forest [7] and molecular sensory science approaches have been comprehensively used to screen the differential markers of jiangxiangxing Baijiu from different production regions. Six key flavor components of jiangxiangxing Baijiu (including ethyl octanoate, ethyl 2-methylpropanoate, propyl acetate, ethyl heptanoate, 2-nonanone and butyl hexanoate) could be effectively traced back to the production region. Moreover, recent research has mostly focused on the aroma expression [5,8,9] of trace components, and there has been relatively little research about their impact on taste and sensation. Therefore, VASE and a multi-dimensional sensory evaluation system (i.e., aroma, taste and sensation) were conducted and combined with a random forest model in this study to clarify the differential markers between flavor types and further reveal the evolutionary relationship of Baijiu flavor types.

In this study, the objectives were (1) to identify and analyze the trace components in 12 flavor types of Baijiu with different pretreatments combined with multiple instruments; (2) to screen differential markers between 12 flavor types of Baijiu by molecular sensomics and a random forest model; and (3) to clarify the relationship between their differential markers and sensory attributes by addition tests and further verify the chemical origin of their sensory characteristics.

## 2. Material and Methods

### 2.1. Sample Collection

In this study, Baijiu produced by large-scale enterprises recognized in the industry was selected, and a total of 22 representative samples were selected (Table 1). Due to the numerous representative brands of nongxiangxing Baijiu, jiangxiangxing Baijiu and qingxiangxing Baijiu, multiple samples were selected. For other flavor types, Baijiu from the most representative manufacturer was selected. These samples were all purchased in the market and stored at 4 °C until further analysis.

### 2.2. Analytical Reagents

Ethanol (chromatographically pure, 99.8%) was obtained from Beijing InnoChem Science & Technology Co., Ltd. (Beijing, China). A C_3_–C_30_ n-alkane mixture (Sigma–Aldrich, Shanghai, China) was used for determination of linear retention indices [10]. Sodium chloride (analytically pure, 99.5%, NaCl) was obtained from Chengdu Colon Chemical Co., Ltd. (Chengdu, China). The internal standard substances (2-ethylbutyric acid, IS1; 4-octanol, IS2; and pentyl acetate, IS3) and the analytical standards employed for the identification and quantitative analysis of the trace components were all purchased from J&K Scientific Company (Beijing, China).

### 2.3. Extraction, Quantification and Identification of Trace Components

#### 2.3.1. Extraction of Trace Components by VASE [4]

A diluted sample (2.0 mL) with a final ethanol concentration of 15.0% *v/v* was placed into a headspace bottle (20.0 mL) with a silicone rubber septum and saturated with sodium chloride (0.4 g). A total of 40.0 μL of mixed internal standards solution (2-ethylbutyric acid, IS1; 4-octanol, IS2; and pentyl acetate, IS3; with a mass concentration of 1000 mg/L) was added. The sorbent pen was inserted into the headspace bottle and formed a seal with the bottle cap liner. A vacuum pump was used to vacuum the bottle to <0.01 atm through the sorbent pen. The vacuum sealing between the lid pad and the sorbent pen allowed the sample to remain in a vacuum state after 30 s, thereby increasing the static diffusion rate and collecting many more headspace components on the adsorbent than at atmospheric pressure. The headspace bottle was equilibrated in extraction system (5600 SPES; Entech Instrument, Simi Valley, CA, USA) for 20 min at a temperature of 50 °C and a speed of 240 r/min. After extracting, the sorbent pen was placed on a cold tray for 20 min for water management, and then it was placed in an isolation sleeve for gas chromatography–mass spectrometry (GC-MS) analysis.

#### 2.3.2. Analytical Conditions for GC-MS

Gas chromatography–mass spectrometry (GC-MS) with a DB-FFAP polar chromatographic column (60 m × 0.25 mm × 0.25 μm; Agilent Technologies, Santa Clara, CA, USA) was used to identify trace components. Each sample was analyzed in three replicates. Every sample (1.0 μL) treated via VASE was injected in spitless mode and analyzed. Helium (99.999%) was used as a carrier gas at a constant flow rate of 1.0 mL/min, and the inlet temperature was 250 °C. Oven temperature was held at 40 °C at first, then raised to 50 °C at a rate of 10 °C/min and held for 10 min, then increased by 3 °C/min up to 80 °C and held for 10 min, and finally increased by 5 °C/min up to 230 °C and held for 10 min. The total run time for each GC-MS analysis was about 73 min. The mass spectrometry (MS) was operated in electron ionization (EI) mode at 70 eV. The temperature of the interface and the ion source were set to 150 and 230 °C, respectively. The identification of trace components was conducted in a full-scan mode. The temperature of the transfer line was 245 °C. The mass range was set from 50 to 450 amu.

#### 2.3.3. Isolation of Trace Components by Liquid–Liquid Extraction (LLE)

The 30 mL sample was diluted to 15% ethanol (*v*/*v*) with saturated salt water and extracted three times with dichloromethane (50 mL each time). The combined organic phase (about 150 mL) was extracted three times using the Na_2_CO_3_ solution (50 mL each time; 0.50 mol/L; pH 10.0) and then washed using 50 mL of the saturated NaCl solution. The combined aqueous phase (about 200 mL) was acidified to pH of 2.0 with HCl (4.0 mol/L) and extracted three times using the freshly distilled dichloromethane (70 mL each time). Next, after being dried over anhydrous Na_2_SO_4_ for 12 h at −20 °C, both fractions were separated and concentrated to 1.5 mL and then blown to 500.0 µL using a nitrogen blowing instrument [11]. A total of 1.0 µL was injected separately for GC-O-MS analysis.

#### 2.3.4. Screening of Trace Components by GC-O-MS

Screening of trace components was carried out through a gas chromatography–olfactometry–mass spectrometry (GC-O-MS) with a DB-WAX polar chromatographic column (60 m × 0.25 mm × 0.25 μm; Agilent Technologies, Santa Clara, CA, USA). The temperature of the olfactory port was kept at 250 °C. The oven temperature was maintained at 40 °C initially, raised to 50 °C at a rate of 10 °C/min, then increased at a rate of 1 °C/min to 70 °C and held for 10 min, and finally increased at a rate of 3 °C/min up to 250 °C and held for 15 min. The temperature of the transfer line was 250 °C and that of the MS ion source was set to 230 °C. MS fragmentation was detected in the electron impact mode (ionization energy of 70 eV) with an acquisition range from *m*/*z* 50 to 450 in full-scan mode [12].

#### 2.3.5. Odor-Specific Magnitude Estimation (Osme)

During the described GC run, the group members held their noses close to the sniffing port, responded to the aroma intensity of the stimulus, and recorded the aroma descriptor, intensity values and retention time. Aroma descriptors were determined through a previous evaluation of the flavor quality of the reference ingredient. Three panelists were familiar with aroma descriptors based on the aroma criteria provided. Strength was judged using a 5-point scale (0 (none), 1 (extremely weak), 2 (weak), 3 (moderate), 4 (strong), and 5 (very strong)). The Osme value of aroma intensity was the average result of the group members.

#### 2.3.6. Identification of Trace Components

The similarity between the mass spectrometry information of each chromatographic peak and the mass spectrometry libraries of the National Institute of Standards and Technology (NIST) and LIQUOR V1.0 (team self-built mass spectra library) was at least 80%. Combined with the results of comparisons of standard components and retention index, this was considered to be the identification method for this component.

### 2.4. Quantification and Odor Active Value/Taste Active Value (OAV/TAV) Calculation of Trace Components

The quantification of target trace components was carried out using the internal standard method combined with the calibration curve method. The reserve solution was prepared in a 70% *v/v* solution and then diluted to a series of concentrations to obtain a working standard solution. IS 1-3 was added to the above working standard solution and all samples. Then, under the same conditions as above, 1.0 µL of Baijiu sample and working standard solution were injected into the GC without diversion. A calibration curve was drawn by plotting the response ratios of the target trace components and their corresponding IS values against their concentrations.

The concentrations of the target trace components were calculated based on the calibration curves. The analytical limits of detection (LOD) of trace components was obtained from the lowest concentrations of the analyte standard solutions based on a signal-to-noise ratio of 3. All analyses were conducted with three replicates.

The odor thresholds for trace components were obtained from previous papers [12,13,14,15], and taste thresholds for trace components were obtained from previous papers [4,16,17,18]. According to the ratio of quantitative results to threshold values [19] (i.e., concentration/threshold), the OAVs/TAVs of trace components were obtained.

### 2.5. Sensory Quantitative Descriptive Analysis (SQDA)

According to the methods reported in related studies, the sensory evaluation team was composed of 10 sensory evaluators (5 males and 5 females, aged from 21 to 28 years) with olfactory experience. The sensory quantitative descriptive analysis method was used to evaluate the sensory attributes of Baijiu samples. The descriptors with higher frequencies were screened out. All sensory evaluators were called together to discuss descriptors until an agreement was reached on their sensory attributes. Sensory attributes were listed based on the description of aroma and taste of Baijiu. Sensory attributes refer to the following: a jiao aroma, grainy aroma, jiang aroma, baked aroma, floral/fruity aroma, sweet aroma, mild aroma, herbal aroma, ethanol aroma, honey aroma, sesame aroma, milky aroma, oily aroma, softness, richness, mellowness, sweetness, hardness, neatness, sourness, harmony, sweet aftertaste, bitter aftertaste, persistence and off-taste. Finally, the evaluators were provided with Baijiu samples (10.0 mL) in glass bottles (25.0 mL) coded with 3-digit numbers and asked to score the sensory intensities using the 5-point scale mentioned above. The sensory evaluation was performed in a sensory panel room at 25 ± 1 °C with a humidity of 35–50%, and each sample was evaluated in triplicate [20].

### 2.6. Flavor Addition Experiments

Addition experiments were conducted to verify the contribution of the differential markers screened through machine learning to the sensory attributes of Baijiu. According to the actual concentration in sample tested, each differential marker was added to the Baijiu sample with lowest concentration in each group. Then, as described in Section 2.5, the sensory attributes of the samples were evaluated by the same evaluators.

### 2.7. Statistical Analysis and Statistical Methods

Statistical analyses were performed using Excel 2021. The random forest models were established using an online website (https://www.metaboanalyst.ca (accessed on 20 December 2023)) to screen for differential markers. The correlation analysis was conducted with Pearson correlation coefficient. Graphics were drawn using an online website (https://www.chiplot.online/ (accessed on 27 December 2023)).

## 3. Results and Discussion

### 3.1. Analysis of Distribution Characteristics of Trace Components

For the purpose of clarifying the distribution of the trace components of different flavor types of Baijiu, VASE-GC-MS was applied comprehensively. As shown (Table S1), a total of 319 trace components were identified from Baijiu of 12 flavor types, including 83 esters, 47 alcohols, 18 acids, five lactones, 15 aldehydes, 26 ketones, 10 acetals, seven sulfur components, five pyrazines, 57 aromatics (containing benzene), 25 alkanes and 21 furans.

**Figure 1:**
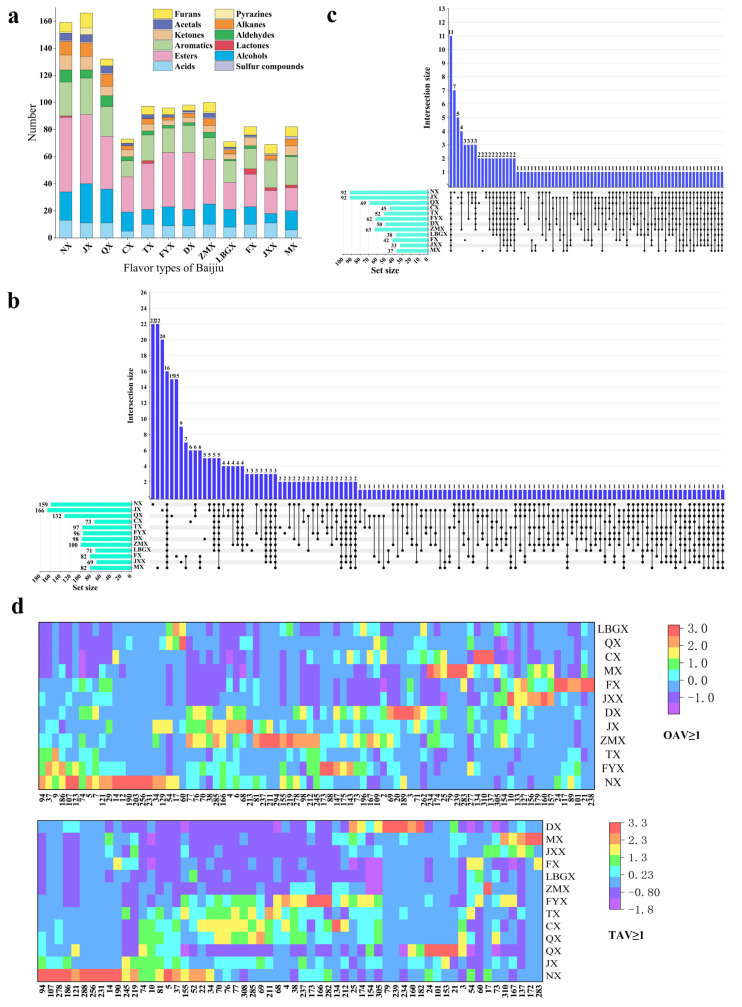
Molecular sensomics analysis of trace components in 12 flavor types of Baijiu. (**a**) Stacked bar chart of the distribution of trace component species. (**b**) UpSet plot of the distribution of trace component species. (**c**) UpSet plot of aroma expression evaluation of trace components. (**d**) Heat map of trace components’ distribution with TAVs ≥ 1 and OAVs ≥ 1.

As presented in Figure 1a,b, trace components in NX, JX and QX were relatively abundant, with 159, 166 and 132 kinds, respectively. There was a consistent phenomenon among the 12 flavor types of Baijiu, i.e., esters, acids, alcohols and aromatics (containing benzene) accounted for the highest proportion. Esters represent the most abundant trace components in Baijiu, formed through three primary pathways: (1) microbial metabolism; (2) lipase-catalyzed esterification reactions; and (3) chemical reactions occurring during the aging process of Baijiu [21]. Additionally, each flavor type of Baijiu possessed its own distinct trace components. Notably, 126 kinds of trace components were identified in only one flavor type of Baijiu (including 22 in NX, 22 in MX, 20 in JX and 15 in QX, respectively), which collectively account for 39.50% of the total identified trace components. Specifically, five pyrazines were identified in JX, potentially contributing to its unique baking flavor. In contrast, 16 kinds of trace components were found across all flavor types, representing 5.02% of the total. Overall, the majority of trace components (60.50%) were commonly found across various flavor types of Baijiu, suggesting similarities in both trace component composition and sensory quality among the different flavor profiles.

### 3.2. Aroma Expression Evaluation of Trace Components

To investigate the aroma expression of the trace components in Baijiu, LLE combined with GC-O-MS and the Osme evaluation method were performed. The results (Table S1) indicated that esters primarily impart fruity and sweet aromas, while acids contribute sour, fatty and cheesy aromas. Alcohols predominantly exhibit bad aromas, such as greasy aromas, although some present fruity and floral characteristics. All pyrazines exhibited a nutty aroma. Moreover, the aroma intensities of the trace components were recorded using the Osme evaluation method. In general, the aroma intensity of acids and esters was higher than that of other trace components, followed by aromatics (containing benzene) and alcohols. As presented in Figure 1c, only 11 kinds of trace components were identified across all flavor types of Baijiu. The aroma expression of other trace components varied among the different flavor types of Baijiu, lacking a consistent pattern. Consequently, further research is required to elucidate the specific aroma contribution of trace components to the flavor profile of Baijiu.

### 3.3. Quantification of Trace Components

In order to clarify the characteristics of trace components in 12 flavor types of Baijiu, according to the above results and trace components with strong flavor expression in previous reports, 119 kinds of trace components were selected and quantified. As shown in Table S2, esters accounted for the highest proportion of all flavor types of Baijiu. Among them, the concentration of ethyl hexanoate was the highest in NX (5794.97 ± 168.35 mg/L), while the concentration of ethyl acetate (11771.56 ± 267.86 mg/L) and ethyl lactate (2488.03 ± 61.29 mg/L) were the highest in JX, and ethyl butanoate had the highest concentration in DX (1262.26 ± 39.85 mg/L). These four esters, which may be the key factors affecting the quality and style of Baijiu, were produced by microbial metabolism [22]. In addition, several trace components were only detected in one flavor of Baijiu. For example, 2,3,5-trimethylpyrazine and 2,3,5,6-tetramethylpyrazine, which usually existed in Baijiu fermented at a high temperature [23], were detected in JX. On the whole, the concentrations of trace components in diverse flavor types of Baijiu were significantly different. The concentrations of higher alcohols in MX were higher, such as 2-methyl-1-propanol and (165.22 ± 17.96 mg/L) and 3-methyl-1-butanol (784.24 ± 13.83 mg/L), which were consistent with those in an existing report [24] and related to the hydrolysis of raw rice when brewing to produce a large number of amino acids [25]. The concentrations of aldehydes and ketones detected in the JX samples were significantly higher than those in other samples, which were determined by JX’s unique geographical location and special brewing materials and processes. Most JX Baijiu is produced in the Chishui river basin. The sorghum grown there is less glutinous and has a high amylopectin content, which can withstand multiple rounds of baking and cooking and forms a unique grain flavor during production [26]. The high concentration of pyrazine compounds (such as 2,6-dimethylpyrazine, 2,3,5,6-tetramethylpyrazine and 2,3,5-trimethylpyrazine) in JX and ZMX was related to the use of high-temperature Daqu (a saccharification and fermentation agent) in their production [27,28]. The concentration distribution characteristics of trace components classified by category were basically the same, such as more esters and acids and less furans, which indicated that different flavor types of Baijiu were largely similar in flavor. However, due to the matrix effect, it was not sufficient to evaluate the contribution of trace components to Baijiu flavor just from their concentrations.

### 3.4. OAVs and TAVs of Trace Components

As mentioned above, the contribution of trace components in Baijiu depended not only on their concentration, but also on the interaction between them and matrix effects [29]. A GC-O-MS analysis was used for identifying aromas without considering matrix effects. Hence, in order to reveal the flavor contributions of the trace components in Baijiu, the odor thresholds [12,13,14,15] and taste thresholds [4,16,17,18] of trace components from the literature were used to estimate their OAVs and TAVs. OAVs or TAVs ≥ one means that the trace components have a direct flavor contribution to the Baijiu, and the larger the OAV/TAV, the more significant its contribution to the overall flavor quality. The overall distribution characteristics for trace components are shown in Figure 1d. As shown, trace components with OAVs ≥ one were mainly concentrated in NX, JX, FYX, TX, DX and ZMX, and these flavor types of Baijiu were more abundant in aroma quality, while trace components with TAVs ≥ one were mainly concentrated in NX, QX, JX, FYX, TX and CX, which were richer in terms of their taste.

As shown in Table S2, a total of 83 kinds of trace components (including 27 esters, 10 alcohols, 11 acids, four aldehydes, six ketones, one acetal, three sulfur components, one pyrazines, 16 aromatics (containing benzene), two alkanes and two furans) with OAVs ≥ one were screened as aroma components. Among these, six components were detected in all flavor types of Baijiu: ethyl acetate, ethyl hexanoate, ethyl octanoate, butanoic acid, octanoic acid and furfural. Esters imparted pleasant fruity and sweet aromas to the Baijiu, while acids not only enhanced its flavor but also served as precursors to the esters. Given their higher OAVs and corresponding aroma intensities, esters and acids were considered the two most important classes of aroma components in Baijiu. Currently, most studies focus on the aroma contribution of trace components, while relatively little research has been conducted on their contributions to taste.

Considering the significance of taste quality in Baijiu and the fact that individuals predominantly choose Baijiu based on their taste preferences, it was imperative to further investigate the taste contribution of trace components in Baijiu. As exhibited in Table S3, a total of 60 kinds of trace components (including 15 esters, 10 alcohols, six acids, one lactone, three aldehydes, five ketones, two acetals, three sulfur components, 11 aromatics (containing benzene), one alkane and three furans) were screened as taste components with TAVs ≥ one. Notably, ethyl acetate, ethyl hexanoate, ethyl octanoate, butanoic acid, 2-methyl-1-propanol, 1-butanol, benzaldehyde and phenethyl alcohol were screened as taste components in all types of Baijiu. All of these except phenethyl alcohol are also frequently screened as aroma components.

In conclusion, a total of 91 aroma and taste components were screened, which were speculated to contribute directly to the Baijiu and may serve as significant markers for distinguishing between flavor types. Notably, some components with lower concentrations, such as 3-methylbutyric acid, 1-octanol, ethyl laurate, 4-methyl phenol, dimethyl trisulfide and furfuryl alcohol, obtained high final OAVs or TAVs due to their lower thresholds. Nevertheless, further research is needed to verify whether these aroma and taste components contribute to the overall flavor profile of Baijiu and to explore their interactions.

### 3.5. SQDA on Twelve Flavor Types of BAIJIU

**Figure 2:**
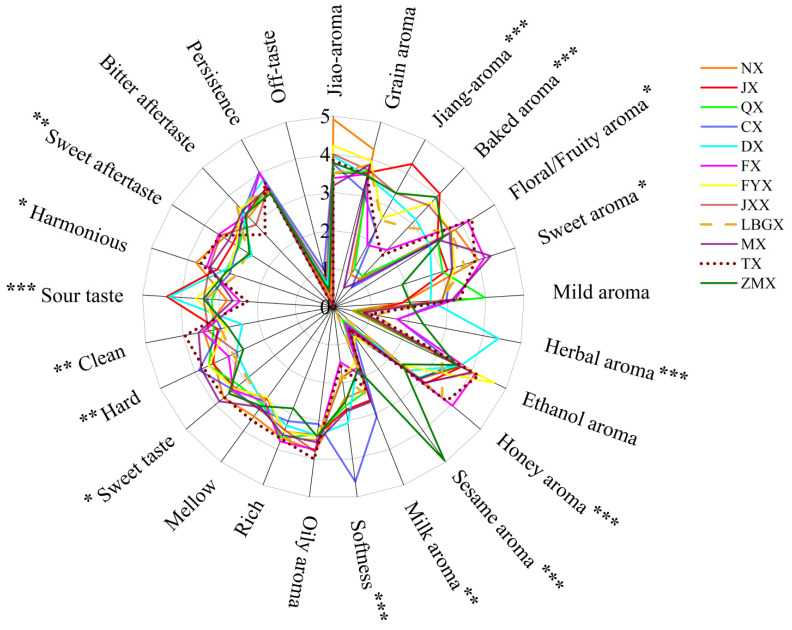
Sensory radar maps of 12 flavor types of Baijiu. The significant differences between samples were represented by *** (*p* < 0.001), ** (0.001 ≤ *p* < 0.01), and * (0.01 ≤ *p* < 0.05).

Based on the SQDA, the sensory attributes of 12 flavor types of Baijiu were evaluated and are shown as sensory radar maps (Figure 2). Overall, there was a great amount of diversity in the sensory profiles between the 12 flavor types of Baijiu, with varying intensities of each sensory attribute across the different flavor types. There was relatively little off-taste recorded for all flavor types, likely due to the representative nature of the Baijiu samples tested. Specifically, JX exhibited characteristics of a jiang aroma and sourness, while NX featured a jiao aroma and sweetness. QX was noted for its distinct grain aroma, and the ethanol aroma was most prominent in MX. In addition, the sensory attributes of other derived flavor types had new features. For instance, DX was characterized by a herbal aroma and sourness, attributed to the incorporation of traditional Chinese medicine during the brewing process. CX had a pronounced oily aroma as a result of the steeping process with chen rou (i.e., fatty pork). It was worth noting that ZMX expressed a prominent sesame aroma, closely linked to the complex production technology used and its ecological environment. However, the relationship between Baijiu flavor types is not understood at the molecular level. Based on this, machine learning could be employed to further analyze the correlation of and difference between Baijiu flavor types.

### 3.6. Correspondence Analysis on the Evolution of Baijiu Flavor Types

#### 3.6.1. Analysis of Similarities (ANOSIM) of 12 Flavor Types of Baijiu

**Figure 3:**
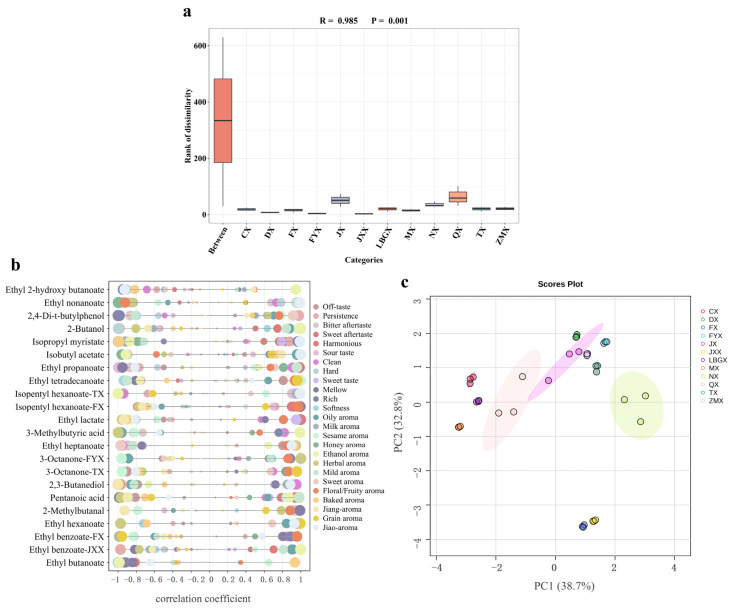
Correspondence analysis of the evolution of Baijiu flavor types. (**a**) ANOSIM between all samples tested and flavor types. (**b**) The relationship between differential markers and sensory attributes based on SQDA. (**c**) The PCA score plot of 12 flavor types of Baijiu by differential markers.

Baijiu is a complex mixture. Even if it is classified according to flavor type, factors such as production region also cause differences in its trace components. In order to minimize the misleading effect of other characteristics and further screen the differential markers between the different flavor types of Baijiu, an analysis of similarities (ANOSIM) was conducted based on the distribution characteristics of trace components. ANOSIM is a non-parametric test used to determine whether the differences between groups (two or more groups) are significantly greater than the differences within groups and, thus, whether the grouping is meaningful [7]. Specifically, the samples with the same flavor type were considered as intra-group samples in the dataset, and the samples were grouped according to flavor type to form inter-group samples. The R-value was used to indicate whether there was a difference between groups, and the *p*-value was used to indicate whether there was a significant difference. The results are shown in Figure 3a, where an R-value of 0.985 indicates that the inter-group difference was greater than the intra-group difference; that is, the difference between flavor types was significantly greater than the difference in Baijiu with the same flavor type. A *p*-value of 0.001 indicated that the inter-group difference was significant. These results fully demonstrated that although there were differences among Baijiu of the same flavor type due to multiple factors such as region and technology, the differences between different flavor types were more significant. Therefore, it was feasible to use reasonable methods to differentiate the flavor types of Baijiu.

#### 3.6.2. Differential Analysis on the Evolution of Baijiu Flavor Types through Machine Learning

The dataset of the trace components in the Baijiu was extensive, and the flavor of the Baijiu was perceived as notably complex [30]. Traditional identification methods, which primarily utilize principal component analyses (PCAs), cluster analyses or similar methods, are often limited in their effectiveness when analyzing small datasets. These methods typically struggle with non-linear relationships and larger datasets. In contrast, machine learning approaches, such as random forest [7] and support vector machine (SVM) models, possess the advantage of being able to uncover potential unknown connections within extensive datasets [31]. Recently, machine learning has been employed to establish identification methods for food based on various treatment techniques [32]. In this study, a random forest analysis (Figure S2) was performed, focusing on the distribution characteristics of trace components. Within 500 decision trees, the trace components were ranked according to their feature importance, ultimately identifying the differential markers between derived and basic flavor types with a high accuracy [33]. Based on this, the Pearson correlation coefficient was calculated to analyze the relationship between the concentrations of differential markers and the scores of the SQDA for Baijiu, as illustrated in Figure 3b. In this figure, 3-octanone-TX and 3-octanone-FYX indicated that 3-octanone was screened in both the TX and FYX groups, a trend that applied to other components as well.

Jianxiangxing (JXX) Baijiu was identified as a flavor type in 1983. Its production technique was inspired by that of JX in the early stage and NX in the late stage, forming sensory features that combined both the JX and NX styles [34,35]. As shown in Figure S2, ethyl benzoate and ethyl butanoate made an important contribution to identifying JXX and its basic flavor types. Among them, in a previous study [36], ethyl benzoate was also identified as a flavor differential marker to distinguish JXX and its basic flavor types. The correlation analysis (Figure 3b) indicated that ethyl butanoate was negatively correlated with an ethanol aroma, softness and persistence, while positively correlated with a baked aroma. Meanwhile, ethyl benzoate was positively correlated with an oily aroma and ethanol aroma but negatively correlated with a bitter aftertaste.

Dongxiangxing (DX) Baijiu was identified as a flavor type in 1986. Its production technique combined Daqu and Xiaoqu (two saccharification fermenters with different production processes) [37], and its flavor contained the characteristics of three flavor types, namely, JX, NX and MX. A feature of DX was that traditional Chinese medicine was added, which provided a comfortable herbal aroma while promoting or inhibiting microorganisms. In terms of its flavor, DX possessed a unique style, with a composite aroma composed of a herbal aroma, ester aroma and ethanol aroma. As shown in Figure S2, pentanoic acid (positively correlated with a mild aroma and sour taste while negatively correlated with mellowness), 2-methylbutanal (positively correlated with mellowness and a bitter aftertaste while negatively correlated with a jiang aroma) and ethyl hexanoate (negatively correlated with a grain aroma while positively correlated with a herbal aroma and harmony) played an important role in distinguishing DX regarding its basic flavor types.

Texiangxing (TX) Baijiu, using whole-grain rice as its brewing material, was identified as a new flavor type in 1988. The unique ratio of flour, wheat bran and distilled grains used in the production of Daqu were important factors that contributed to the formation of the TX’s style [38,39]. TX simultaneously possessed the characteristics of three basic flavor types (referring to JX, NX and QX). The results (Figure 3b) show that 3-octanone, isopentyl hexanoate and ethyl heptanoate, which had the highest contribution, were all negatively correlated with a bitter aftertaste.

Fengxiangxing (FX) Baijiu has a long history, but its flavor type was not officially determined until 1992. The raw material for its Daqu is consistent with that for QX, while the production technology used is similar to that of NX, meaning FX possesses the sensory characteristics of both QX and NX [40]. Figure S2 shows that 3-methylbutyric acid (positively correlated with an ethanol aroma while negatively correlated with an oily aroma), ethyl lactate (positively correlated with a sweet taste and a sweet aftertaste), isopentyl hexanoate (positively correlated with a floral/fruity aroma, mellowness, harmony and a sweet aftertaste), ethyl tetradecanoate (positively correlated with a floral/fruity aroma and grain aroma while negatively correlated with softness) and ethyl benzoate (positively correlated with a floral/fruity aroma while negatively correlated with a milky aroma and grainy aroma) were identified as major contributors to the classification of FX and its basic aroma types. In particular, the low concentration of ethyl lactate in FX was related to its short fermentation cycle [41], which may have a positive impact on the sweetness of Baijiu.

Zhimaxiangxing (ZMX) Baijiu was officially determined as an independent flavor type in 1995, and its production technology is similar to that of JX. Due to the less humid and hot climate in the north compared to the south, the microorganisms produced during the accumulation process are not abundant enough [42], resulting in Baijiu with a prominent sesame aroma. As shown in Figure S2, ethyl propanoate (negatively correlated with a jiang aroma and mellowness) made the greatest contribution to distinguishing between ZMX and JX.

Chixiangxing (CX) Baijiu was identified in 1996 and is derived from MX. Its processing includes simultaneous saccharification and fermentation. Specifically, it differs from MX in its soaking process, which uses chen rou (i.e., fatty pork) [43]. Chixiang has a unique aroma that combines the basic aroma of Baijiu (such as an ester aroma, floral aroma, etc.) with the mature aroma of chen rou [44]. The results (Figure S2) showed that isobutyl acetate (positively correlated with a jiao aroma and oily aroma while negatively correlated with a honey aroma and ethanol aroma) played an important role in distinguishing between CX and MX.

Laobaiganxiangxing (LBGX) Baijiu, whose acid/ester ratio is basically the same as that of QX and whose concentration of ethyl acetate is also higher, was once considered as QX. After a long period of study, it was found that there are significant differences in the distribution characteristics of the trace components between LBGX and QX [45]. It was not until 2005 that LBGX was officially recognized as a separate flavor type. The classification and feature selection of QX and LBGX were performed using random forest models, and the results (Figure S2) show that isopropyl myristate (positively correlated with a jiao aroma and mellowness while negatively correlated with an oily aroma and baked aroma) made an important contribution to their classification.

Fuyuxiangxing (FYX) Baijiu is derived from NX, JX and QX, and has a combination of a pre-NX flavor, a mid-QX flavor and a post-JX flavor [46]. It was officially identified as an independent flavor type in 2005. The main characteristic of its production technology is that unbroken grains are used as raw materials and saccharified using Xiaoqu to cultivate microorganisms, while Daqu is added for fermentation in a cellar [47]. There were five kinds of differential markers selected by the random forest models (Figure S2), namely, 2-butanol (positively correlated with an ethanol aroma and richness), ethyl 2-hydroxy butanoate (negatively correlated with a jiao aroma, mellowness, sweetness and hardness), 3-octone (positively correlated with a herbal aroma while negatively correlated with a sesame aroma), ethyl nonanoate (positively correlated with a jiao aroma while negatively correlated with softness) and 2,4-di-tert-butylphenol (positively correlated with a sweet aroma and persistence, while negatively correlated with harmony and richness).

In summary, the random forest model was used to classify and select the features of the derived and basic flavor types. The 19 differential markers with the highest contribution to the classification of these types were selected based on the accuracy ranking of each group, including 11 esters, two alcohols, two acids, one aldehyde, one ketone and two aromatics (containing benzene). Based on this, a PCA analysis was carried out on the different flavor types with 19 selected differential markers to evaluate whether they can distinguish the flavor types of Baijiu. The results (Figure 3c) show that the total variance was 71.5% (PC1 was 38.7%, and PC2 was 32.8%), which indicated that the 19 differential markers screened could effectively distinguish Baijiu of different flavor types. However, the influence of these differential markers for the corresponding flavor type of Baijiu on their sensory attributes still needed to be further verified by flavor addition tests.

### 3.7. Correlation Analysis Based on Flavor Addition Experiments

**Figure 4:**
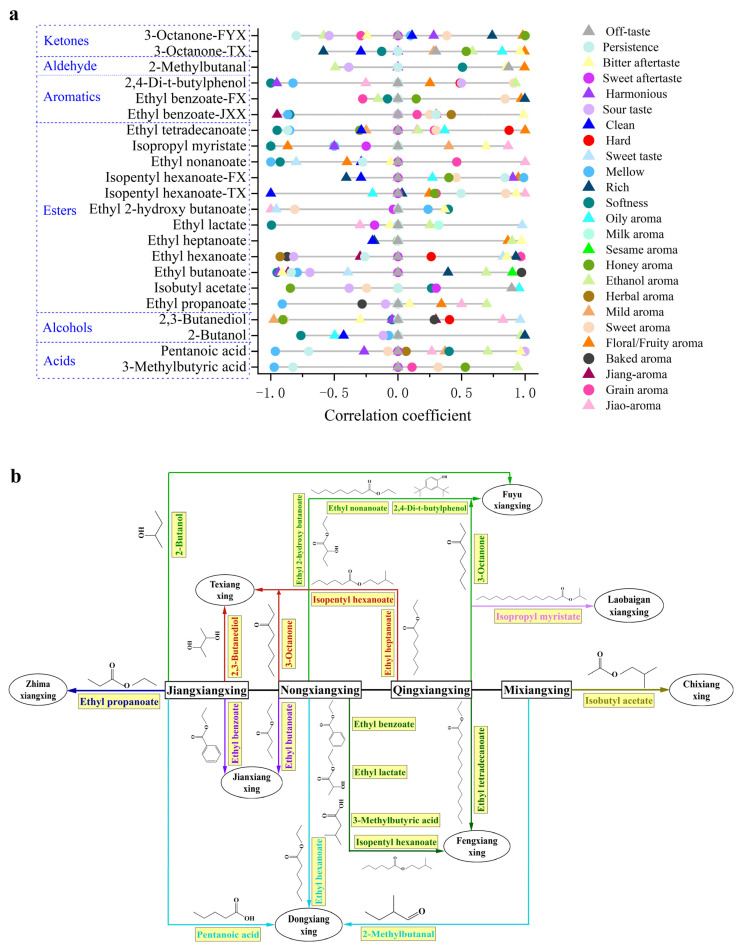
Differential markers of 12 flavor types. (**a**) The relationship between differential markers and sensory attributes based on flavor addition experiments. (**b**) The evolutionary pathways of Baijiu flavor types based on differential markers.

On the basis of the research discussed above, each differential marker was added to the corresponding Baijiu with the lowest concentration in each group, and the sensory attributes were scored based on the SQDA. Then, we visualized the relationship between the sensory attributes and differential markers by a Pearson correlation analysis. As shown in Figure 4a, acids were negatively correlated with mellowness and persistence and positively correlated with an ethanol aroma and bitter aftertaste. In detail, pentanoic acid had a strong positive correlation with a sour taste, which was the prominent sensory attribute of DX according to its sensory radar map, and 3-methylbutyric acid had a strong negative correlation with mellowness and an ethanol aroma. The contribution of alcohols to sensory attributes was distinctive in different Baijiu matrices. Specifically, 2-butanol, a differential marker between FYX and JX, had a strong positive correlation with an ethanol aroma, whose sensory intensity in FYX was much higher than in JX. Meanwhile, 2,3-butanediol was positively correlated with a jiao aroma, which was consistent with previous research [12].

As the most abundant class of components, most esters had a positive correlation with a floral/fruity aroma and a negative correlation with softness. Ethyl butanoate, ethyl lactate, ethyl nonanoate, isopropyl myristate and ethyl tetradecanoate were observed to have a strong negative correlation with softness. Meanwhile, some esters had a strong positive correlation with a jiao aroma, such as ethyl hexanoate, isopentyl hexanoate, ethyl nonanoate and isopropyl myristate. In addition, ethyl propanoate, as a differential marker between JX and ZMX, promoted a sesame aroma. It was speculated that many components jointly affect the sesame aroma of Baijiu. Propionic acid acts as a precursor for ethyl propionate, directly influencing its synthesis. The production of ethyl propionate is positively correlated with the acid production capacity of propionic acid-producing bacteria, such as *Propionibacterium jensenii*, during the brewing process [48]. Therefore, effectively monitoring and managing the levels of these bacteria are crucial for optimizing the flavor profile of Baijiu. According to the above-mentioned research, isobutyl acetate is a differential marker for distinguishing CX and MX, and the result of the addition experiments showed a strong positive correlation between isobutyl acetate and an oil aroma, which may be due to the difference in the production technology of CX and MX. Significantly, isopentyl hexanoate made various sensory contributions to different flavor types of Baijiu, such as the positive contribution to jiao-aroma in JXX while negative in FX. It was preliminarily supposed that isopentyl hexanoate had synergistic or inhibitory effects in different Baijiu matrices. Similarly, ethyl 2-hydroxybutyrate was not considered to have a direct contribution to the senses according to the OAV/TAV analysis, but the result of the addition experiments showed a strong negative correlation with a jiao aroma, a sweet aroma and a sweet taste, which may also have synergistic or inhibitory effects.

For aromatics (containing benzene), positive correlations with differential markers were found for attributes of a floral/fruity aroma and sweet aroma, which was consistent with previous research [49]. Of note, ethyl benzoate was found to have the highest score for a floral/fruity aroma followed by a sweet aroma. According to the above conclusions, ethyl benzoate was considered as a differential marker both in the JXX and FX groups, and its sensory contribution in these two flavor types was unequal. Specifically, it was positively correlated with a floral/fruity aroma in the FX group and negatively correlated with a jiang aroma in the JXX group. Although ethyl benzoate was only considered to have a direct contribution to aroma but not to taste according to the OAV/TAV analysis, it was found that ethyl benzoate was correlated with softness, richness, mellowness and a bitter aftertaste. Additionally, 2,4-di-t-butylphenol had a strong positive correlation with sweetness and an ethanol aroma, which were the prominent sensory attributes of FYX. The research found that the unique brewing environment of the FYX-Baijiu-producing area fosters a distinct microbial community. The weak acid mud cellar serves as the optimal growth environment for caproic acid and butyric acid bacteria, which play a key role in aroma production. The interactions between the enzyme production and metabolic activities of these microorganisms contribute to the sweetness and ethanol aroma of Baijiu, enhancing its overall flavor profile [50].

Aldehydes and ketones exhibited similar contributions to the sensory attributes of Baijiu, representing a strong positive correlation with a floral/fruity aroma, a sweet aroma and a bitter aftertaste. For 3-octanone, a differential marker identified both in the TX and FYX groups, its contribution to a floral/fruity aroma was consistent. Compared to NX, TX had a more prominent floral/fruity aroma according to above sensory radar maps; indeed, the addition of 3-octanone greatly enhanced the intensity of its floral/fruity aroma. Moreover, 3-octanone also showed a strong positive correlation with a honey aroma in the FYX group.

To sum up, the results of this study are illustrated in Figure 4b, demonstrating the evolutionary pathways of these differential markers and their corresponding flavor types. The sensory characteristics of Baijiu across different flavor types were formed through the combined action of various trace components, mainly consisting of esters, acids, alcohols, aromatics (containing benzene), aldehydes and ketones. For example, esters, aromatics (containing benzene), aldehydes and ketones had a positive impact on the Baijiu’s floral/fruity aroma and sweet aroma, while alcohols predominantly affected the Baijiu’s ethanol aroma. The Baijiu’s sour taste was primarily due to acids, and the Baijiu’s mellowness was mainly due to acids and certain esters. Additionally, differential markers with lower OAVs and TAVs may still exert an indirect impact on the sensory attributes of Baijiu, with their sensory contributions varying across different Baijiu matrices, probably due to synergistic or inhibitory effects. Based on these findings, the differential markers between various flavor types of Baijiu effectively explained their sensory differences. Finally, the addition experiments proved that the contribution of the 19 differential markers (including 3-methylbutyric acid, pentanoic acid, 2-butanol, 2,3-butanediol, ethyl propanoate, isobutyl acetate, ethyl butanoate, ethyl hexanoate, ethyl heptanoate, ethyl lactate, ethyl 2-hydroxy butanoate, isopentyl hexanoate, ethyl nonanoate, isopropyl myristate, ethyl tetradecanoate, ethyl benzoate, 2,4-di-t-butylphenol, 2-methylbutanal and 3-octanone) to the overall sensory attributes of Baijiu varied significantly. These were verified as key differential markers and effectively elucidated the evolutionary pathways of Baijiu flavor types. In previous studies, most of these differential markers were identified as key flavor active components in various flavor types of Baijiu. For instance, ethyl lactate, ethyl butanoate, ethyl hexanoate and 3-methylbutyric acid were highlighted in FYX [51], while ethyl hexanoate, ethyl lactate and ethyl butanoate were prominent in JXX [36]. Additionally, ethyl tetradecanoate, ethyl butanoate, isopentyl hexanoate, ethyl lactate and ethyl hexanoate were noted in MX [52]. These findings confirmed that the differential markers were critical components of the characteristic flavor of Baijiu. Furthermore, this study bridged the gap in our understanding of the relationship between these key components and the differences in Baijiu flavor types.

## 4. Conclusions

In total, 319 trace components were identified in Baijiu across 12 flavor types using GC-O-MS and GC-MS. Among them, 91 trace components were further recognized as aroma and taste components owing to their relatively high OAVs and TAVs in all samples. Ultimately, 19 differential markers (including 3-methylbutyric acid, pentanoic acid, 2-butanol, 2,3-butanediol, ethyl propanoate, isobutyl acetate, ethyl butanoate, ethyl hexanoate, ethyl heptanoate, ethyl lactate, ethyl 2-hydroxy butanoate, isopentyl hexanoate, ethyl nonanoate, isopropyl myristate, ethyl tetradecanoate, ethyl benzoate, 2,4-di-t-butylphenol, 2-methylbutanal and 3-octanone) were screened and validated between the derived and basic flavor types of Baijiu using random forest models combined with a PCA, which could effectively reveal the evolutionary pathways of Baijiu flavor types. The key differential markers exhibited varying degrees of influence on the sensory characteristics of Baijiu across different flavor types. Of note, differential markers with lower OAVs and TAVs may still exert an indirect impact on the sensory attributes of Baijiu, and their sensory contributions could vary across different matrices, probably due to synergistic and inhibitory effects. This study provides a theoretical foundation for the scientific and standardized expression of Baijiu flavor quality and supports the digital and intelligent development of the traditional brewing industry.

## Figures and Tables

**Table 1:** Information on Baijiu samples.

No.	Abbreviation	Flavor Types	Ethanol Concentration/%*v*/*v*
1	NX	nongxiangxing	52
2	NX	nongxiangxing	39
3	NX	nongxiangxing	52
4	NX	nongxiangxing	52
5	NX	nongxiangxing	42
6	JX	jiangxiangxing	53
7	JX	jiangxiangxing	53
8	JX	jiangxiangxing	53
9	JX	jiangxiangxing	53
10	JX	jiangxiangxing	46
11	QX	qingxiangxing	53
12	QX	qingxiangxing	53
13	QX	qingxiangxing	52
14	CX	chixiangxing	53
15	TX	texiangxing	52
16	FYX	fuyuxiangxing	54
17	DX	dongxiangxing	54
18	ZMX	zhimaxiangxing	53
19	LBGX	laobaiganxiangxing	67
20	FX	fengxiangxing	52
21	JXX	jianxiangxing	42
22	MX	mixiangxing	52

## Data Availability

The original contributions presented in the study are included in the article/Supplementary Materials; further inquiries can be directed to the corresponding author.

## Supplementary Materials

The following supporting information can be downloaded at: https://www.mdpi.com/article/10.3390/foods13193034/s1; Figure S1. Schematic diagram of the vacuum-assisted sorbent extraction (VASE). Figure S2. The results of random forest analysis. Table S1. Trace components identified from Baijiu samples of 12 flavor types by VASE-GC-MS and LLE-GC-O-MS. Table S2. The concentration and OAVs/TAVs of trace components in Baijiu of 12 flavor types.

## Abbreviations

QX: qingxiangxing; NX, nongxiangxing; JX, jiangxiangxing; MX, mixiangxing;CX, chixiangxing; TX, texiangxing; FYX, fuyuxiangxing; DX, dongxiangxing; ZMX, zhimaxiangxing; LBGX, laobaiganxiangxing; FX, fengxiangxing; JXX, jianxiangxing.

VASE: vacuum-assisted sorbent extraction; RF, random forest; GC-MS, gas chromatography–mass spectrometry; LLE, liquid–liquid extraction; GC-O-MS, gas chromatography–olfactometry–mass spectrometry; Osme, odor specific magnitude estimation; OAV, odor active value; TAV, taste active value; SQDA, sensory quantitative descriptive analysis; ANOSIM, analysis of similarities.
