# Supplementary material for: Molecular Sensomics Combined with Random Forest Model Can Reveal the Evolution of Flavor Type of Baijiu Based on Differential Markers
Source: Foods. 2024 Sep 24;13(19):3034. doi: 10.3390/foods13193034 (PMC11476331; doi:10.3390/foods13193034)
Supplement: Supplementary file 1 [file foods-13-03034-s001.zip › Supplementary Figures.pdf]

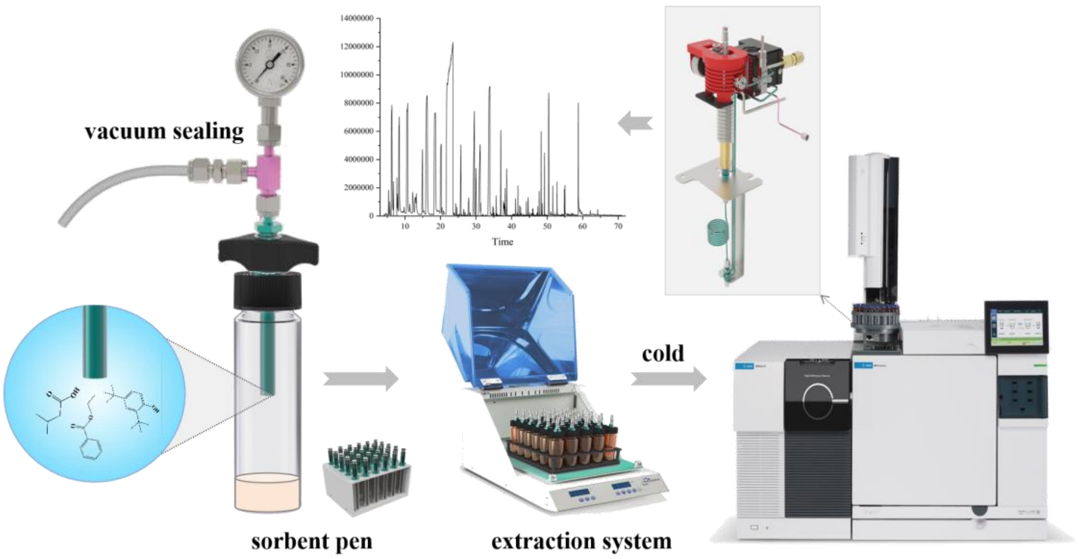

Figure S1. Schematic diagram of the vacuum-assisted sorbent extraction (VASE).

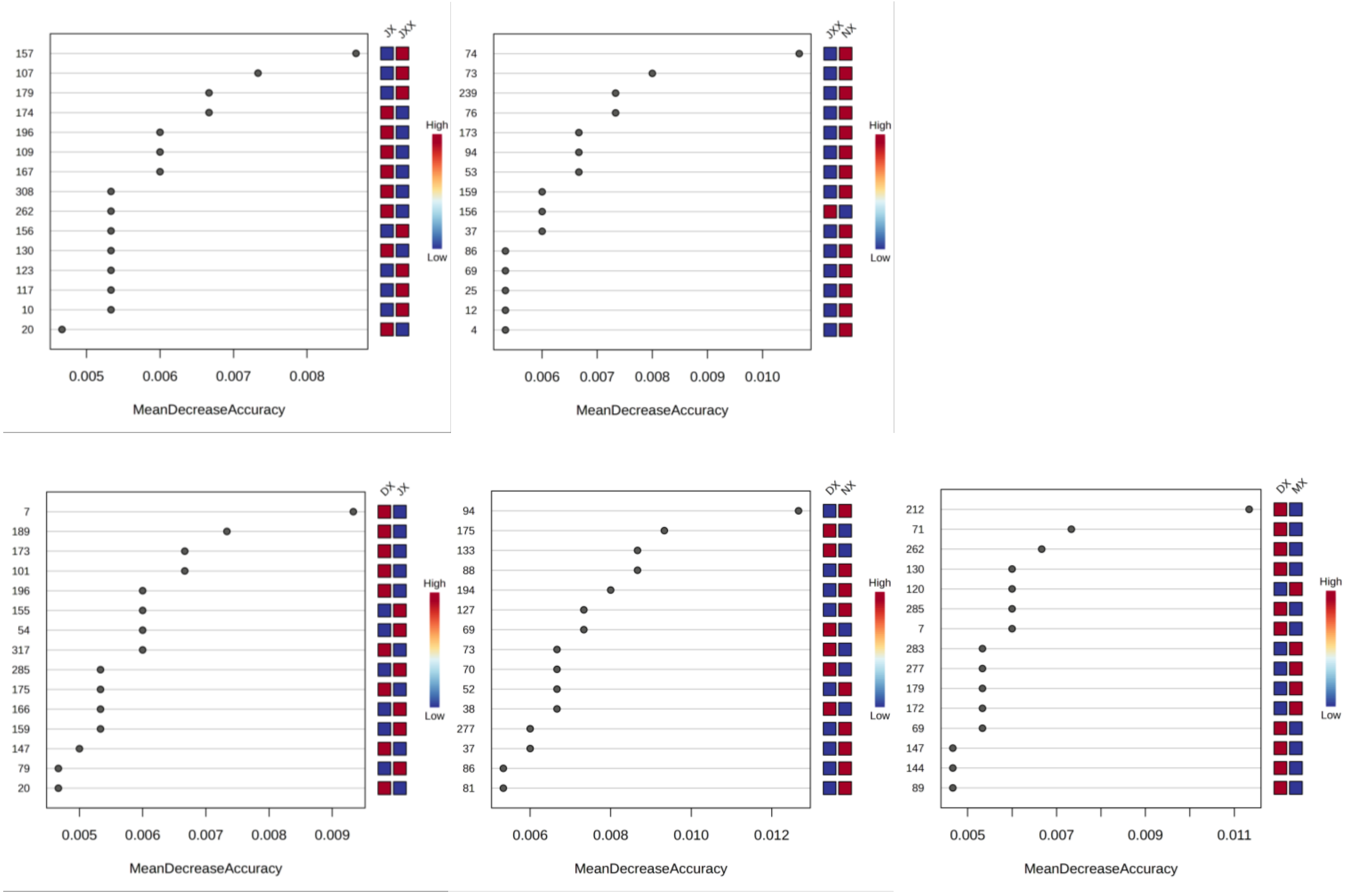

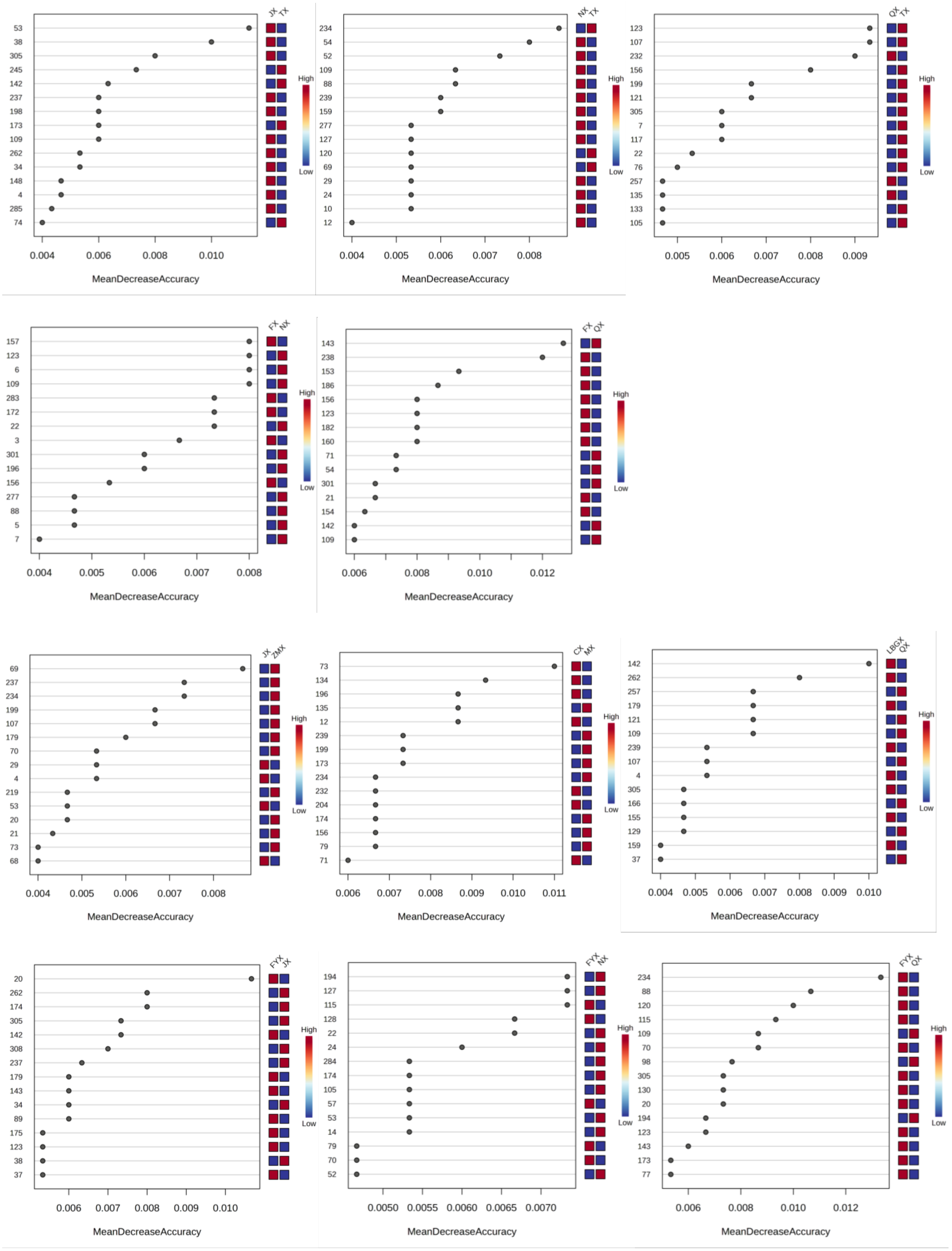
JX TX
53 38 305 245 142 237 198 173 109 262 34 148 4 285 74
0.004 0.006 0.008 0.010
MeanDecreaseAccuracy
High Low
NX TX
234 54 52 109 88 239 159 277 127 120 69 29 24 10 12
0.004 0.005 0.006 0.007 0.008
MeanDecreaseAccuracy
High Low
QX TX
123 107 232 156 199 121 305 7 117 22 76 257 135 133 105
0.005 0.006 0.007 0.008 0.009
MeanDecreaseAccuracy
High Low
FX NX
157 123 6 109 283 172 22 3 301 196 156 277 88 5 7
0.004 0.005 0.006 0.007 0.008
MeanDecreaseAccuracy
High Low
FX QX
143 238 153 186 156 123 182 160 71 54 301 21 154 142 109
0.006 0.008 0.010 0.012
MeanDecreaseAccuracy
High Low
JX ZMX
69 237 234 199 107 179 70 29 4 219 53 20 21 73 68
0.004 0.005 0.006 0.007 0.008
MeanDecreaseAccuracy
High Low
CX MX
73 134 196 135 12 239 199 173 234 232 204 174 156 79 71
0.006 0.007 0.008 0.009 0.010 0.011
MeanDecreaseAccuracy
High Low
LBGX QX
142 262 257 179 121 109 239 107 4 305 166 155 129 159 37
0.004 0.005 0.006 0.007 0.008 0.009 0.010
MeanDecreaseAccuracy
High Low
FYX JX
20 262 174 305 142 308 237 179 143 34 89 175 123 38 37
0.006 0.007 0.008 0.009 0.010
MeanDecreaseAccuracy
High Low
FYX NX
194 127 115 128 22 24 284 174 105 57 53 14 79 70 52
0.0050 0.0055 0.0060 0.0065 0.0070
MeanDecreaseAccuracy
High Low
FYX QX
234 88 120 115 109 70 98 305 130 20 194 123 143 173 77
0.006 0.008 0.010 0.012
MeanDecreaseAccuracy
High Low

Figure S2. The results of random forest analysis. In the legend, blue represented a negative correlation between variables and flavor types, while red represented a positive correlation. The contribution of variable features to the accuracy of inter group classification was expressed as Mean Difference Accuracy (MDA), and the higher the value, the greater the contribution of variables to sample classification.
